# Long-Term Moderate Increase in Medium-Chain Fatty Acids Intake Enhances Muscle Metabolism and Function in Mice

**DOI:** 10.3390/ijms26094126

**Published:** 2025-04-26

**Authors:** Ziwei Zhang, Cong Wu, Shuo Wang, Yishan Tong, Jiapeng Huang, Chuwen Xue, Tiehan Cao, Katsuhiko Suzuki

**Affiliations:** 1 Graduate School of Sport Sciences, Waseda University, Tokorozawa 359-1192, Japan; 2 Faculty of Sport Sciences, Waseda University, Tokorozawa 359-1192, Japan

**Keywords:** medium-chain fatty acids, grip strength, exercise tolerance, muscle regeneration, muscle differentiation, protein catabolism

## Abstract

Medium-chain fatty acids (MCFAs) refer to a mixture of fatty acids typically composed of 6 to 12 carbon atoms. The unique transport and rapid metabolism of MCFAs provide more clinical benefits than other substrates, such as long-chain fatty acids. Although many studies have shown that MCFAs may improve exercise capacity and muscle strength, applications have mainly been limited to low doses. This study explores the effects of high-dose MCFA intake on muscle strength and exercise endurance. Mice were fed high-fat diets containing 30, 35, and 40% (*w*/*w*) MCFAs for 12 weeks, and measurements of grip strength and submaximal endurance exercise capacity were conducted to evaluate muscle function. Results showed that compared to the 30% MCFAs group, the absolute grip strength in the 35 and 40% MCFAs groups significantly increased; in terms of endurance performance, the 35% MCFAs group showed a significant increase compared to the 40% MCFAs group. These results were mainly achieved by promoting muscle regeneration and differentiation and inhibiting the expression of the ubiquitin-proteasome pathway. This study demonstrates that moderately increasing MCFA intake can improve the effects of obesity-induced muscle atrophy. However, excessive intake may reduce the impact of improvement.

## 1. Introduction

**Figure 1:**
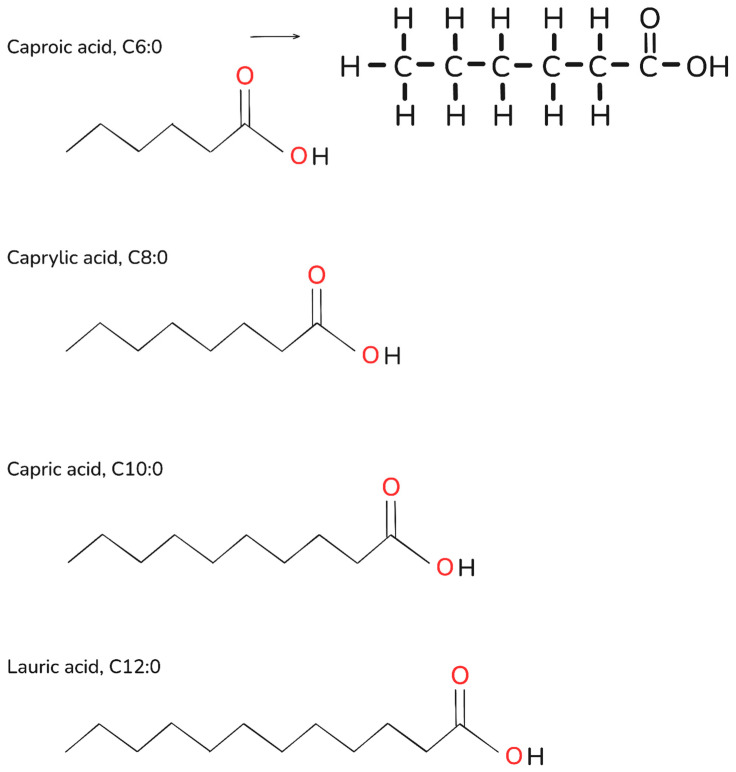
Chemical structures of medium-chain fatty acids (MCFA). This figure was drawn using Excalidraw (https://excalidraw.com/) accessed on 26 March 2024.

Fatty acids are important biological molecules in structure formation, signal transduction, and energy storage [1]. Medium-chain fatty acids (MCFA) refer to a mixture of fatty acids typically consisting of 6 to 12 carbon atoms (Figure 1). Medium-chain triglycerides (MCT) are biologically processed esters with glycerol molecules attached to MCFA. Commercial MCFA products mainly comprise C8:0 and C10:0 [2]. MCFA is found in natural substances, and its unique properties have been studied for decades [3].

Compared to the main lipid compounds of long-chain fatty acids (LCFA; 13-21 carbon atoms), MCFA exhibits significant absorption, transport, and metabolism differences due to the shorter carbon chains. MCFA is directly absorbed into the portal vein and mainly travels through the plasma as free fatty acids (FFAs) complexed with plasma albumin [4]. The binding efficiency of free MCFA to plasma albumin depends on the chain length, with the binding equilibrium constant increasing as the chain length increases [4]. In the liver, MCFA is directly absorbed into hepatocytes, undergoing β-oxidation, ketogenesis, or lipogenesis [5,6]. Their relatively short carbon chains allow MCFA to diffuse through the plasma membrane, accelerating their metabolism [5]. In liver mitochondria, MCFA enters through diffusion and is readily metabolized. However, the transport of LCFA depends on proteins and relies on binding to carnitine (the rate-limiting step) [7]. Therefore, MCFA is absorbed and metabolized much faster than LCFAs, serving as a quicker and more direct energy source.

Due to their unique and rapid metabolic characteristics, MCFA possesses multiple metabolic effects. In addition to being an energy source, MCFA can play new roles in cellular metabolic signaling through receptor-mediated and non-receptor-mediated pathways [8]. They are considered potential treatments for diseases such as diabetes, obesity, epilepsy, Alzheimer’s disease, and other metabolic disorders [6,9].

Some scholars have proposed that MCFA intake can improve exercise capacity and muscle strength. Guo et al. [10] showed that dietary supplementation with 1% lauric acid (C12) can improve aerobic exercise endurance and muscle strength in sedentary mice. Fushiki et al. [11] indicated that a long-term 80 g/kg MCT diet can improve mice’s swimming ability. Nosaka et al. [12] indicated that for amateur athletes, daily intake of food containing 6 g MCT over 14 days can inhibit the increase in blood lactate concentration and RPE during moderate-intensity exercise and prolong subsequent high-intensity exercise time at levels higher than those achieved by consuming LCT-containing food. Fukazawa et al. [13] indicated that long-term intake of a ketogenic diet containing 315.25 g/kg MCT and relatively high carbohydrates can reduce body weight and abdominal fat mass and enhance skeletal muscle keratolysis capacity induced by endurance training in male rats. The results of Abe et al. [14] indicated that a daily intake of 6 g MCT per person can enhance muscle strength and function in frail elderly individuals and improve their ability to perform daily activities. Nutr et al. [15] found that supplementing dinner with 6 g MCT, leucine-rich amino acids, and cholecalciferol can improve muscle strength and function in frail elderly individuals. Kojima et al. [16] indicated that combining aerobic exercise and MCT intake (6 g per person per day) may effectively prevent muscle strength decline and increase muscle strength, as this can improve muscle energy production. This can help maintain the health of middle-aged and elderly individuals at high risk of frailty and sarcopenia.

As dietary molecules from natural sources, MCFAs demonstrate value beyond their role as an energy source. Despite their broad application prospects, existing research results remain heterogeneous and are mainly limited to low-dose or low-proportion applications [17,18,19]. The effects of high-dose intake of MCFAs within safe limits on muscle strength and exercise endurance still lack sufficient research.

Previous research has demonstrated that mice fed a high-fat diet supplemented with 30% MCFA (equivalent to 60 g MCFA/kg feed) exhibited multiple advantages compared to the obese control group, including obesity prevention, reduced visceral fat content, and preservation of lean body mass [17]. This dosage (60 g/kg) represents one of the highest levels in current MCFA research, with its safety well documented [20]. Based on these findings, we established three MCFA dosage groups within the safe range of 60–80 g/kg [21].

Given that numerous studies have indicated that MCFA intake can enhance muscular strength, we hypothesized that MCFA administration would improve muscle function in mice. To test this hypothesis, we investigated the chronic effects of the aforementioned three MCFA doses on muscle function, encompassing both muscular strength and exercise endurance. Additionally, to elucidate the underlying molecular mechanisms, we analyzed plasma biomarkers associated with tissue damage and energy metabolism while evaluating the expression of mRNAs involved in muscle development pathways.

## 2. Results

### 2.1. Systemic Metabolic and Physiological Responses to MCFA Intake

#### 2.1.1. Food and Water Intake and Body Weight Changes During MCFA Administration

**Figure 2:**
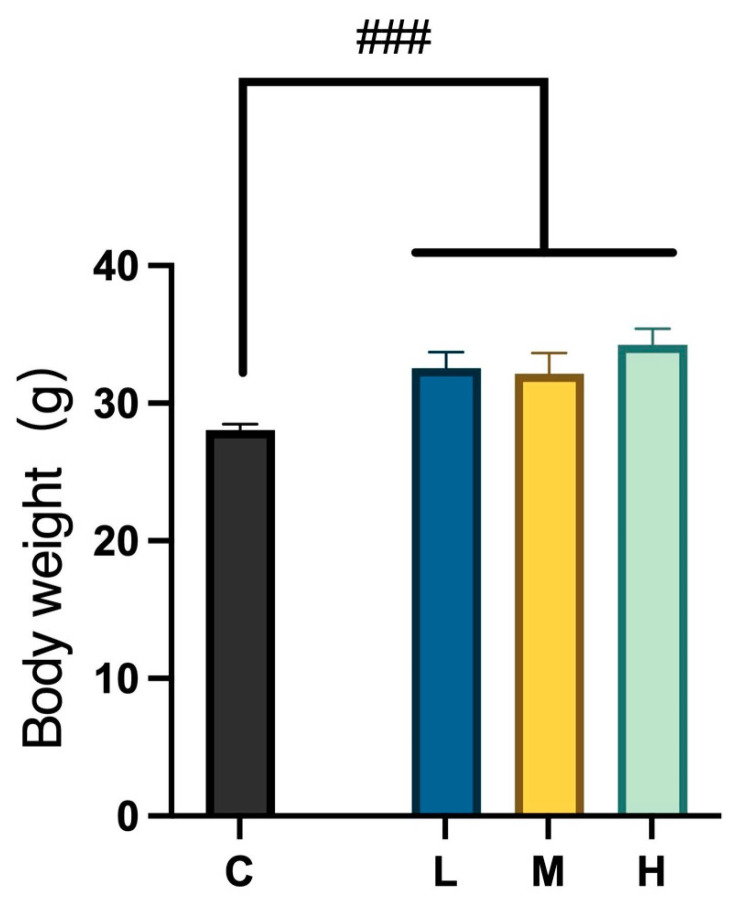
Comparison of body weights between the control and three treatment groups after 12 weeks of intervention; ### *p* < 0.001 (unpaired *t*-test). Data are presented as mean ± standard deviation (SD).

As shown in Table 1, there were no statistically significant differences in water intake and food consumption among the four experimental groups. However, due to the varying fat content in different foods, there were notable weight differences between the control group and the three MCFA treatment groups (Figure 2).

The weekly body weight changes of the experimental animals are provided in the Supplementary Materials (Table S1).

#### 2.1.2. Changes in Organ and Tissue Mass Following MCFA Intake

We measured the organ or tissue weights of the mice and calculated the organ weight to body weight ratios. As shown in Table 2, after 12 weeks of MCFA administration, there were no significant differences in the absolute and relative weights of the heart, liver, kidneys, brown fat, adipose tissue, and muscle tissue that we collected. Compared with the control group (group C), the weights of brown adipose tissue, epididymal white adipose tissue, retroperitoneal fat, and mesenteric fat were significantly increased in all three MCFA treatment groups.

#### 2.1.3. Analysis of Visceral Function and Energy Metabolites

**Figure 3:**
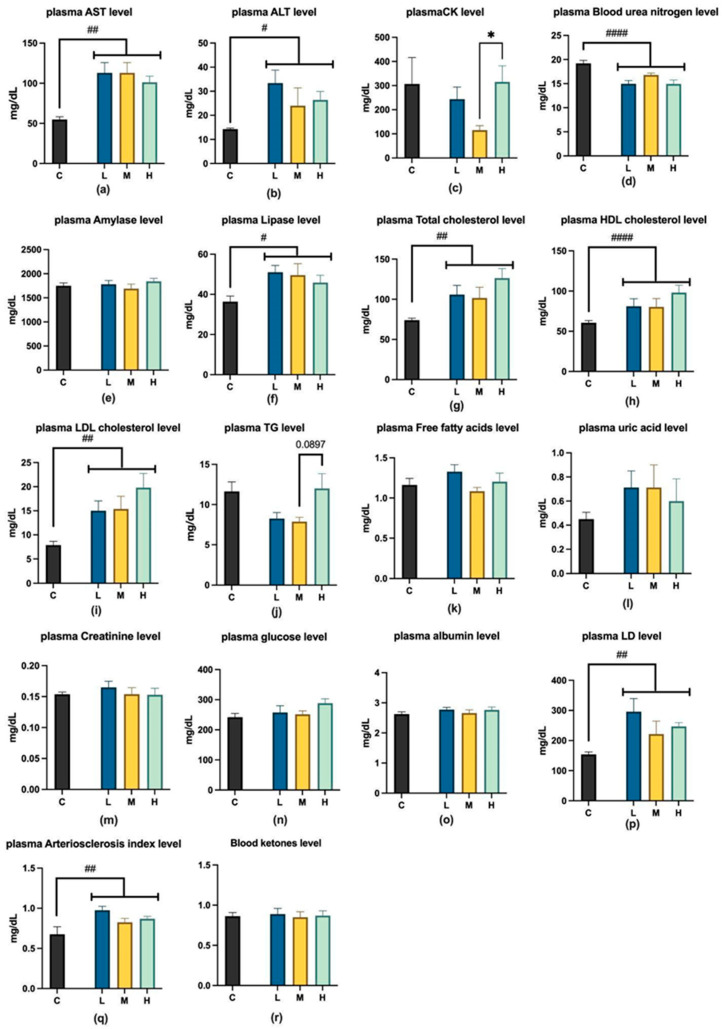
Plasma levels of (**a**) AST, (**b**) ALT, (**c**) CK, (**d**) blood urea nitrogen, (**e**) amylase, (**f**) lipase, (**g**) total cholesterol, (**h**) HDL, (**i**) LDL, (**j**) TG, (**k**) free fatty acids, (**l**)uric acid, (**m**) creatinine, (**n**) glucose, (**o**) albumin, (**p**) LD, (**q**) arteriosclerosis index, and (**r**) ketones. AST, aspartate aminotransferase; ALT, alanine aminotransferase; CK, creatine kinase; LDH, lactate dehydrogenase; LDL, low-density lipoprotein cholesterol; HDL, high-density lipoprotein cholesterol; C group, low-fat diet control group; L group, high-fat diet with low-proportion MCFA (30%, *w*/*w*) treatment group; M group, high-fat diet with medium-proportion MCFA (35%, *w*/*w*) treatment group; and H group, high-fat diet with high-proportion MCFA (40%, *w*/*w*) treatment group. Data are presented as mean ± standard deviation (SD); * *p* < 0.05, compared with the L group by one-way ANOVA with post hoc test; # *p* < 0.05, ## *p* < 0.01, and #### *p* < 0.0001; *t*-test was performed between the three LMH experimental groups and group C.

Figure 3 shows the effects of 12-week MCFA treatment on blood biochemical levels. Among the three experimental groups, no significant differences were observed in UA, BUN, and Cr, which are related to kidney injury; AST, ALT, and ALB, which are associated with liver injury; and plasma amylase and plasma lipase, which are linked to pancreatic damage. However, significant differences were observed in CK values between LD and CK, which are associated with muscle damage. The CK values in group M significantly decreased compared to group H. Furthermore, group M showed a decreasing trend in TG values compared to group H among the metabolic regulation-related indicators (TC, HDL, LDL, TG, FFA, AIP, GLU, and blood ketones).

### 2.2. MCFA Effects on Skeletal Muscle Function

#### 2.2.1. Assessment of Forelimb Grip Strength

**Figure 4:**
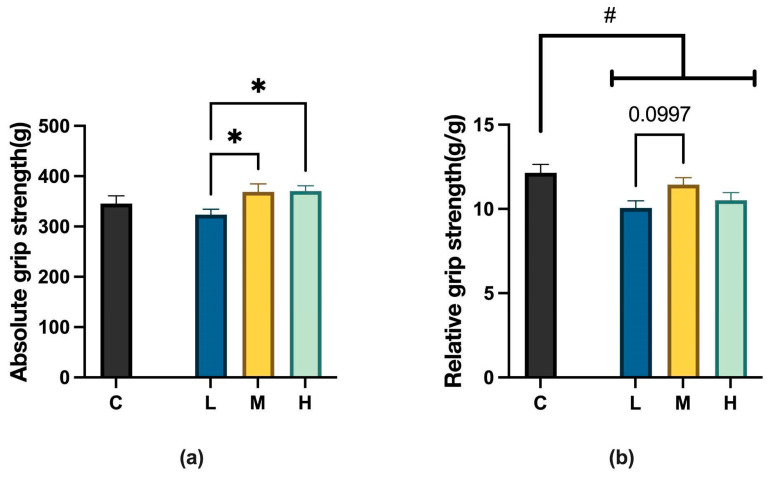
(**a**) Comparison of absolute grip strength in C57BL/6NCrSlc mice. L vs. M, * *p* < 0.05; L vs. H, * *p* < 0.05 (one-way ANOVA with post hoc test). (**b**) Comparison of relative grip strength in C57BL/6NCrSlc mice. L vs. M, *p* = 0.0997(one-way ANOVA with post hoc test). C vs. LMH, # *p* < 0.05 (unpaired *t*-test). C group, low-fat diet control group; L group, high-fat diet with low-proportion MCFA (30%, *w*/*w*) treatment group; M group, high-fat diet with medium-proportion MCFA (35%, *w*/*w*) treatment group; and H group, high-fat diet with high-proportion MCFA (40%, *w*/*w*) treatment group. Data are presented as mean ± standard deviation (SD).

After 12 weeks of MCFA administration, the absolute grip strength of mice in groups C, L, M, and H reached 345.75 ± 43.77 g, 323.38 ± 31.45 g, 369.13 ± 43.87 g, and 370.70 ± 32.52 g, respectively; the relative grip strength of the four groups reached 12.13 ± 1.42 g/g, 10.06 ± 1.18 g/g, 11.44 ± 1.17 g/g, and 10.51 ± 1.42 g/g, respectively. Regarding the absolute grip strength of the three treatment groups, the groups M and H showed significant improvement compared to the group L, with the M group showing a trend of improvement compared to the group H. As for the relative grip strength of the three treatment groups, the group M showed a trend of improvement compared to group L, as shown in Figure 4.

#### 2.2.2. Evaluation of Endurance Exercise Capacity

We evaluated the effects of MCFA administration on maximum exercise time and submaximal endurance exercise capacity in mice at 12 weeks. The test was designed as a blinded assessment to reduce bias, and the experimenters could not determine the time required to reach fatigue. The maximum exercise times for the C, L, M, and H groups were 105.33 ± 38.83 min, 58.33 ± 15.72 min, 62.83 ± 13.17 min, and 44.38 ± 6.50 min, respectively. The submaximal endurance exercise capacities for the four groups were 36,755.22 ± 18,055.53 g·m, 17,201.90 ± 7853.68 g·m, 19,617.66 ± 4978.03 g·m, and 11,270.40 ± 3778.33 g·m, respectively.

**Figure 5:**
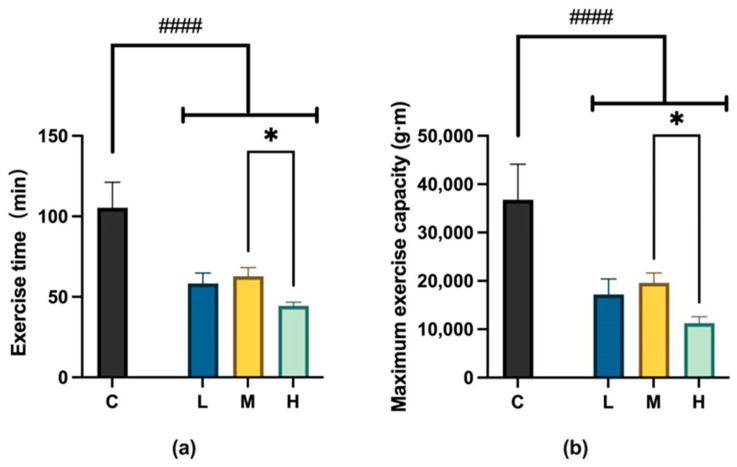
(**a**) Maximum exercise time. M vs. H, * *p* < 0.05 (one-way ANOVA with post hoc test). C vs. LMH, #### *p* < 0.0001 (unpaired *t*-test). (**b**) Submaximal endurance exercise capacity. H, * *p* < 0.05 (one-way ANOVA with post hoc test). C vs. LMH, #### *p* < 0.0001 (unpaired *t*-test). C group, low-fat diet control group; L group, high-fat diet with low-proportion MCFA (30%, *w*/*w*) treatment group; M group, high-fat diet with medium-proportion MCFA (35%, *w*/*w*) treatment group; and H group, high-fat diet with high-proportion MCFA (40%, *w*/*w*) treatment group. Data are presented as mean ± standard deviation (SD).

Regarding the three treatment groups, although the establishment of the obesity model led to a decrease in exercise capacity in all three experimental groups, the decline was most pronounced in the H group. The H group significantly reduced maximum exercise time and submaximal endurance exercise capacity compared to the M group. Meanwhile, there were no significant differences in maximum exercise time and sub-maximum endurance exercise capacity between the groups L and H as well as between the groups L and M. This also indicates that the M group experienced the most minor decrease in exercise capacity and was the least affected by obesity, as shown in Figure 5.

### 2.3. MCFA Regulation of Muscle Satellite Cell Activity

**Figure 6:**
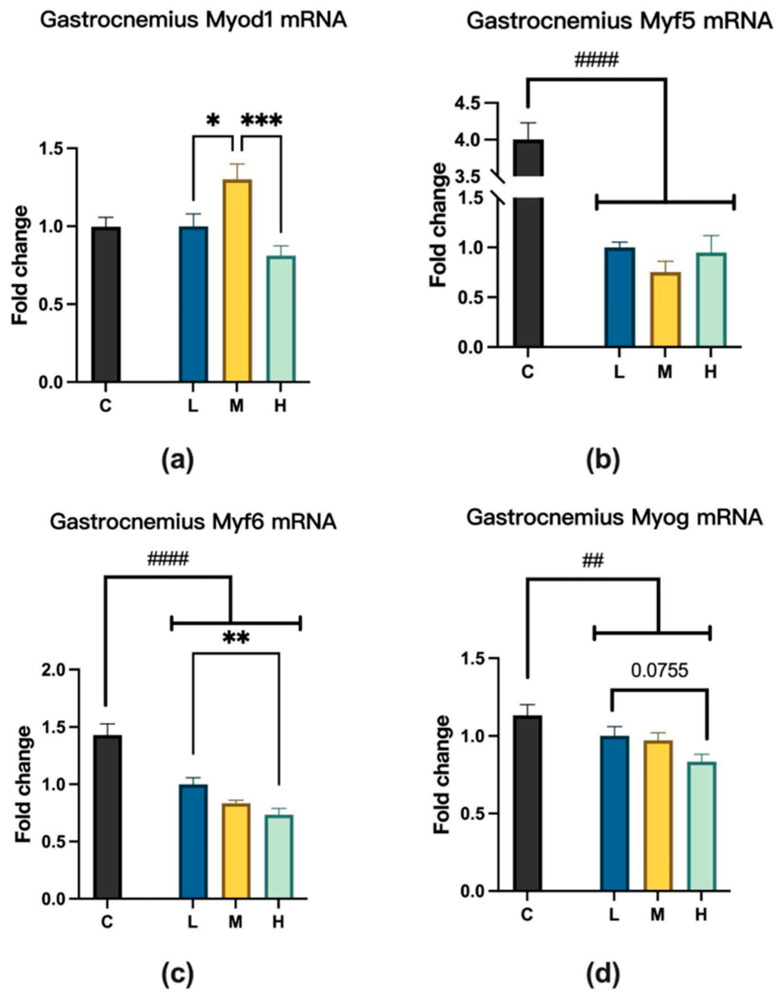
(**a**) Myod1 expression, L vs. M, * *p* < 0.05; M vs. H, *** *p* < 0.001 (one-way ANOVA with post hoc test). (**b**) Myf5 expression, C vs. LMH, #### *p* < 0.0001 (unpaired *t*-test). (**c**) Myf6 expression, L vs. M, ns; L vs. H, ** *p* < 0.01; (one-way ANOVA with post hoc test). C vs. LMH, #### *p* < 0.0001 (unpaired *t*-test). (**d**) Myog expression, L vs. H, *p* = 0.0755; (one-way ANOVA with post hoc test). C vs. LMH, ## *p* < 0.01 (unpaired *t*-test). C group, low-fat diet control group; L group, high-fat diet with low-proportion MCFA (30%, *w*/*w*) treatment group; M group, high-fat diet with medium-proportion MCFA (35%, *w*/*w*) treatment group; and H group, high-fat diet with high-proportion MCFA (40%, *w*/*w*) treatment group. Data are presented as mean ± standard deviation (SD).

Following MCFA administration, we analyzed the expression of genes related to myogenesis in the gastrocnemius muscle to elucidate the underlying mechanisms contributing to improving grip strength. Among the three experimental groups, the expression of myogenic differentiation 1 (Myod1) in the group M was significantly higher than the other two groups; regarding the expression of myogenic factor 6 (Myf6), H showed a significant decrease compared to group L; concerning the expression of myogenin (Myog), the group L showed an increasing trend compared to group H. However, MCFA administration did not affect the mRNA levels of myogenic factor 5 (Myf5), as shown in Figure 6.

### 2.4. Myosin Heavy-Chain Isoform Changes After MCFA Treatment

#### 2.4.1. Myosin Heavy Chain mRNA Expression

**Figure 7:**
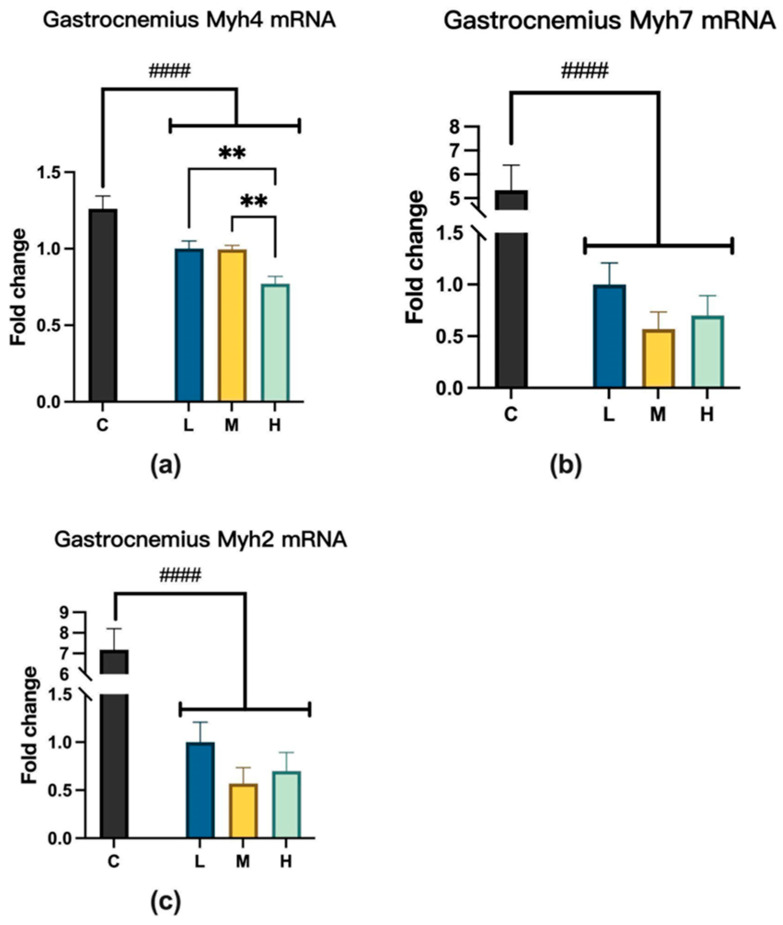
(**a**) Myh4 expression, L vs. H, ** *p* < 0.01; M vs. H, ** *p* < 0.01 (one-way ANOVA with post hoc test). C vs. LMH, #### *p* < 0.0001 (unpaired *t*-test). (**b**) Myh7 expression, C vs. LMH, #### *p* < 0.0001 (unpaired *t*-test). (**c**) Myh2 expression, C vs. LMH, #### *p* < 0.0001 (unpaired *t*-test). C group, low-fat diet control group; L group, high-fat diet with low-proportion MCFA (30%, *w*/*w*) treatment group; M group, high-fat diet with medium-proportion MCFA (35%, *w*/*w*) treatment group; and H group, high-fat diet with high-proportion MCFA (40%, *w*/*w*) treatment group. Data are presented as mean ± standard deviation (SD).

For myosin heavy chain 4 (Myh4) expression, groups L and M were significantly higher than group H. However, MCFA administration did not affect myosin heavy chain 7 (Myh7) and myosin heavy chain 2 (Myh2) mRNA levels, as shown in Figure 7.

#### 2.4.2. Myosin Heavy-Chain Protein Levels

**Figure 8:**
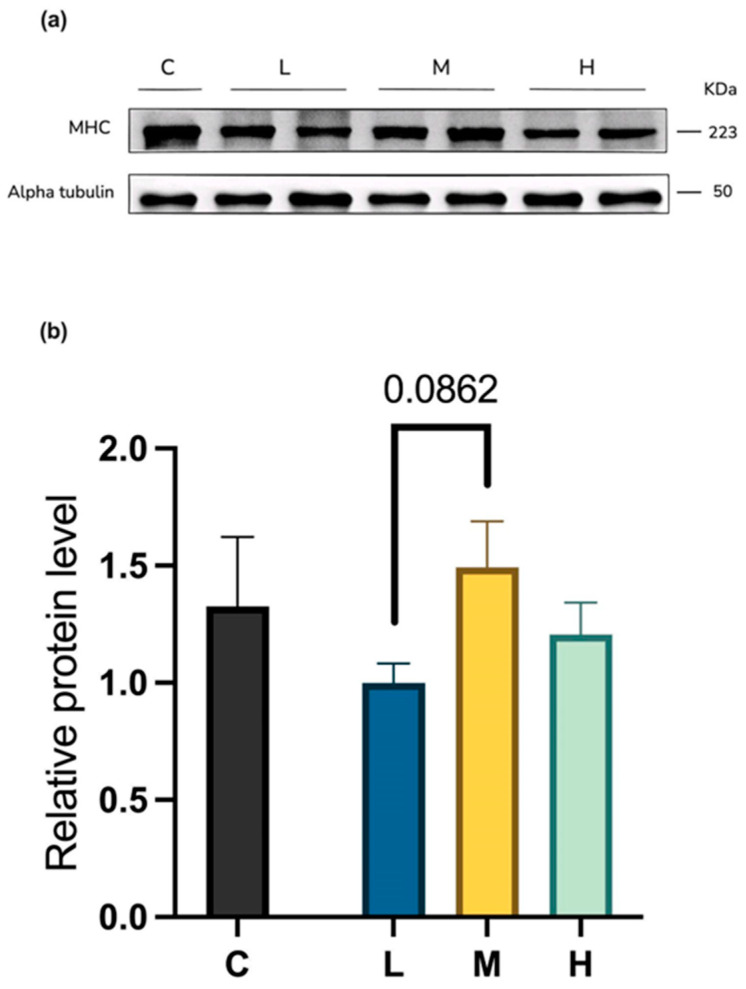
Representative Western blot (**a**) and quantification (**b**) of myosin heavy chain (MHC) in the gastrocnemius muscle of mice after long-term MCFA treatment. L vs. M, *p* = 0.0862; (one-way ANOVA with post hoc test). L group, high-fat diet with low-proportion MCFA (30%, *w*/*w*) treatment group; M group, high-fat diet with medium-proportion MCFA (35%, *w*/*w*) treatment group; and H group, high-fat diet with high-proportion MCFA (40%, *w*/*w*) treatment group. Data are presented as mean ± standard deviation (SD).

Figure 8 presents Western blot data comparing myosin heavy-chain (MHC) expression across four groups. The results indicate a trend towards higher MHC levels in the group M compared to the group L mice, though this increase lacked statistical significance.

### 2.5. MCFA Effects on the Muscle Protein Degradation Pathway

**Figure 9:**
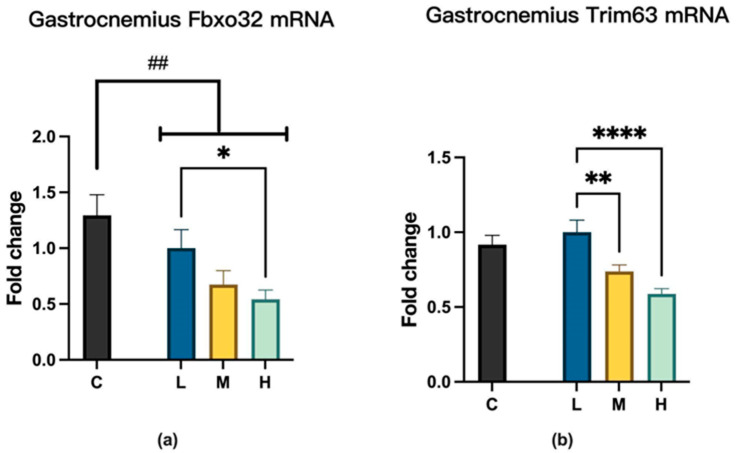
(**a**) Fbxo32 expression, L vs. H, * *p* < 0.05 (one-way ANOVA with post hoc test). C vs. LMH, ## *p* < 0.01. (**b**) Trim63 expression, L vs. M, ** *p* < 0.01; L vs. H, **** *p* < 0.0001 (one-way ANOVA with post hoc test). C group, low-fat diet control group; L group, high-fat diet with low-proportion MCFA (30%, *w*/*w*) treatment group; M group, high-fat diet with medium-proportion MCFA (35%, *w*/*w*) treatment group; and H group, high-fat diet with high-proportion MCFA (40%, *w*/*w*) treatment group. Data are presented as mean ± standard deviation (SD).

Among the three experimental groups, compared to mice in the group L, 12 weeks of MCFA administration reduced the expression of F-box protein 32 (Fbxo32 or ATROGIN1) in the group H and decreased the expression of tripartite motif-containing 63 (Trim63 or MuRF1) in both groups M and H, as shown in Figure 9.

### 2.6. Analysis of Muscle Fiber Size Following MCFA Administration

**Figure 10:**
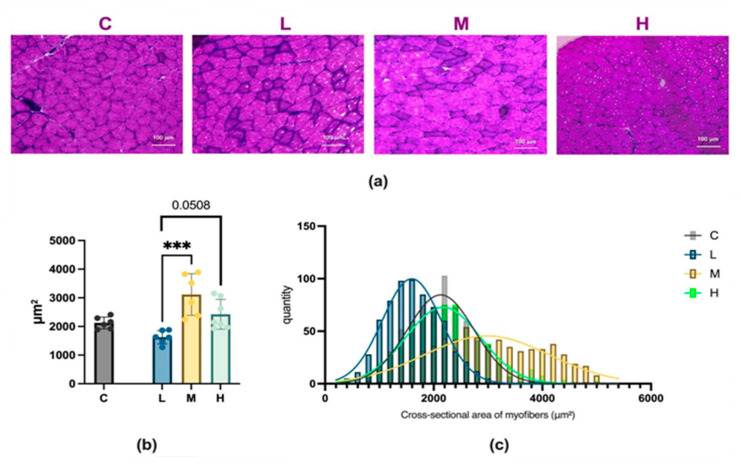
Effects of 12-week MCFA administration on gastrocnemius muscle fiber cross-sectional area (CSA) (H&E). (**a**) Representative microscopic H&E staining of gastrocnemius muscle tissue (20×). (**b**) Mean fiber CSA, L vs. M, *** *p* < 0.001; L vs. H, *p* = 0.0508 (one-way ANOVA with post hoc test). (**c**) Relative frequency distribution of muscle fiber size in gastrocnemius muscle. C group, low-fat diet control group; L group, high-fat diet with low-proportion MCFA (30%, *w*/*w*) treatment group; M group, high-fat diet with medium-proportion MCFA (35%, *w*/*w*) treatment group; and H group, high-fat diet with high-proportion MCFA (40%, *w*/*w*) treatment group. Data are presented as mean ± standard deviation (SD).

Histological assessment of gastrocnemius muscle tissue showed that the average cross-sectional area of muscle fibers reached 2122.11 ± 206.18 μm^2^ in the group C, 1623.69 ± 232.29 μm^2^ in the group L, 3149.85 ± 790.65 μm^2^ in the group M, and 2451.62 ± 571.79 μm^2^ in the group H. Among the three experimental groups, compared to the mice in group L, mice in group M showed a significant increase in the average cross-sectional area of muscle fibers; mice in group H showed a trend of increased average cross-sectional area of muscle fibers, as shown in Figure 10.

## 3. Discussion

To elucidate the potential mechanisms behind the improvement in grip strength and exercise capacity after MCFA treatment, we analyzed the expression of muscle generation-related genes in the gastrocnemius muscle. The gastrocnemius muscle contains a mixture of Type I and Type II fibers as a primary motor muscle with significant metabolic activity. Specifically, Type IID fibers account for approximately 51.5%, Type IIA for 22.7%, Type IIB for 18.2%, and slow-twitch Type I fibers for about 15.7% [22]. This composition makes it an ideal representative tissue for studying muscle metabolism and function. Moreover, the large volume of the gastrocnemius provides sufficient tissue samples for multiple analyses while ensuring sampling consistency between experimental groups.

**Figure 11:**
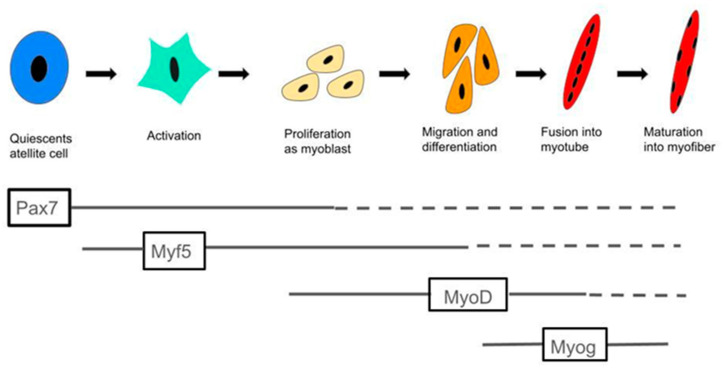
MRFs play a key role in muscle regeneration. Quiescent satellite cells express Pax7. Upon injury, they activate, express *Myf5*, proliferate as myoblasts, and then express *MyoD*. *MyoD*, the primary MRF, regulates differentiation. MyoD-positive cells exit the cell cycle, express myogenin, differentiate, and fuse into myotubes with central nuclei. *Mrf4* expression promotes myotube maturation, forming myofibers with peripheral nuclei. This process demonstrates the importance of MRFs in muscle regeneration. This figure was drawn using Excalidraw “(https://excalidraw.com/) (accessed on 26 March 2024)”.

Muscle regulatory factors (MRFs) are specific helix-loop-helix transcription factors, including *Myod1*, *Myf5*, *Myog*, and *Myf6*. These factors are significantly upregulated during myogenesis and serve as key nodes in myogenic information pathways [23,24], which are essential in directing stem cell differentiation towards myogenic lineages. Myf5 and Myod1 are mainly involved in the early stages of myogenesis and can induce the transformation of non-muscle cells (e.g., fibroblasts) into muscle cells [25,26,27]. Comparatively, Myog and Myf6 act at later stages to promote myotube formation and maturation [26,28]. This process, as shown in Figure 11, reveals the synergistic role of MRFs in the development of muscle tissue, playing a key role in the transition from stem cells to mature myofibers.

Studies have shown that MCFA intake can increase muscle weight [10], improve athletes’ exercise capacity [12,29], and increase muscle strength in the elderly [14,16]. However, research on the effects of MCFA intake on MRFs is still scarce. The concentration-dependent changes in the expression of myogenic factors observed in this study suggest that the M group may most effectively promote the expression of *Myod1*, a key regulatory factor involved in early myogenic commitment. In contrast, the H group appeared to suppress Myf6 and Myog, which may indicate that excessive concentrations inhibit late-stage differentiation or myotube maturation. *MyoD* plays a vital role in muscle tissue as a key myogenic transcription factor [30,31]. During muscle regeneration, *MyoD* expression regulates several muscle-specific genes. These genes include developmental myosin heavy chain (MHCd, present only in regenerating fibers) and myogenin [32,33,34]. These MyoD-regulated genes are essential for muscle formation and growth.

We found that medium to high doses of MCFA administration significantly increased *Myod1* expression in mice, suggesting that MCFA may enhance muscle development or regeneration processes by upregulating myogenic factors. These results indicate that MCFA could be used as a supplement to improve muscle function under sedentary or muscle atrophy conditions. The decrease in *Myf6* and *Myog* expression in the group H may be due to side effects caused by MCFA intake approaching the upper limit of safe intake in this group. This again demonstrates the importance of moderate MCFA consumption. This is also consistent with Astrup et al.’s research, which showed that excessive intake of saturated fatty acids increases the risk of cardiovascular disease (CVD) [35].

Next, we observed the *MYH2*, *MYH4*, and *MYH7* expression levels. These proteins belong to the myosin heavy-chain (MYH) family, and studies have shown that MCFA intake can restore MYH expression levels to normal in mice with myocardial injury [36]. While total MYH expression remained unchanged across treatment groups, a notable reduction in *MYH4* expression was detected in the H group. Since *MYH4* is predominantly associated with fast-twitch glycolytic fibers, this result may imply a potential shift in muscle fiber type under excessive MCFA exposure, further highlighting the regulatory importance of moderate MCFA intake.

However, it should be noted that muscle strength improvement may result from interactions between multiple regulatory factors. The increase in Myod1 induced by medium to high doses of MCFA may be just one potential factor. Further research is needed to investigate other possible mechanisms, such as assessing muscle mass and pathways regulating muscle protein synthesis.

Improvements in grip strength and exercise capacity are related to enhanced muscle development or regeneration and are closely associated with the degree of muscle protein degradation [37]. In this study, our findings suggest that long-term MCFA treatment inhibits muscle protein degradation, as reflected by the decreased mRNA expression of the muscle atrophy marker *Fbxo32 (Atrogin1)* in the H group and a significant reduction in *Trim63 (MuRF1)* expression in both the M and H groups. These two genes are key components of the ubiquitin-proteasome pathway, suggesting a potential suppressive effect of MCFA on proteolysis. These results are consistent with the results of Mori et al. [38].

*Trim63 (MuRF1)* and muscle atrophy *Fbxo32(ATROGIN1)* are E3 ubiquitin ligases that play key roles in the ubiquitin-proteasome system, tagging proteins for degradation; they are consistently upregulated under atrophy conditions, indicating that they are part of a typical response to atrophy [39]. Substantial evidence suggests that MCFA intake can alleviate sarcopenia in the elderly [14,16] or mitigate muscle weakness [15]. These improvements are attributed to their inhibitory effects on *Fbxo32* and *Trim63* expression in skeletal muscle, thereby reducing muscle degradation and improving muscle function [40]. These results suggest that MCFA can prevent muscle protein degradation by decreasing the expression levels of the ubiquitin-proteasome system. This mechanism may be beneficial for maintaining muscle mass and promoting subsequent muscle repair. However, these findings are limited to the mRNA expression stage. Future research will require more molecular biology experimental techniques to elucidate the details of MCFA’s effects on muscle repair, regeneration, and adaptation.

Histological and Western blot analyses further revealed that among the three MCFA treatment groups, the M group exhibited enhanced muscle protein synthesis and increased muscle fiber thickness compared to the L group. In contrast, the H group did not significantly increase myosin heavy-chain protein expression or muscle fiber cross-sectional area. Functionally, the M group demonstrated significant improvement in muscle performance relative to the L group, whereas the H group exhibited a diminished effect compared to the M group.

This may suggest that medium to high proportions of medium-chain fatty acid intake can, to some extent, improve muscle atrophy caused by obesity [41], indicating the potential for muscle adaptation, hypertrophy, and functional improvement. However, appropriate intake is crucial. If the intake is excessive, it may reduce the improvement effects.

Despite these interesting findings, our study has several limitations. First, the lack of an obesity control group limited our ability to comprehensively assess MCFA’s effects on obesity-related muscle atrophy. Future studies should adopt more rigorous methodological planning by incorporating appropriate control groups. Second, considering that sex hormones may affect muscle growth and metabolism, it was only conducted in male mice and did not explore the effects of MCFA on mice of different genders. These limitations provide new directions for future research.

## 4. Materials and Methods

### 4.1. Experimental Animals and Ethical Approval

Male C57BL/6NCrSlc mice (*n* = 34, 8 weeks old) were purchased from Japan SLC Corporation (Machida City, Tokyo). Mice were allowed to adapt to the environment for two weeks before the start of the formal experiment. Two animals were housed together in a cage (27 × 17 × 13 cm) in a controlled environment under a light–dark cycle (12 h of light (08:00–20:00)). The experimental procedure followed the Guiding Principles of Animal Care and Use of the Academic Research Ethics Review Committee of Waseda University and was approved (A23-123).

### 4.2. Diet Composition and Experimental Groups

The control group was fed a low-fat diet (containing 4.3% (*w*/*w*)), while the three treatment groups were fed diets containing 20.0% (*w*/*w*) fat with gradually increasing MCFA content for 12 weeks. The feed composition is shown in Table 3 [17].

Thirty-four mice were randomly divided into four groups: a low-fat diet control group (C, *n* = 8) and three MCFA treatment groups. The MCFA treatment groups consisted of a high-fat diet with low-proportion MCFA (30%, *w*/*w*) group (L, *n* = 8, serving as the baseline for MCFA treatment comparisons), a high-fat diet with medium-proportion MCFA (35%, *w*/*w*) group (M, *n* = 8), and a high-fat diet with high-proportion MCFA (40%, *w*/*w*) group (H, *n* = 10). Mice were observed every week, and changes in food intake, water consumption (distilled water), and body weight were recorded weekly.

### 4.3. Assessment of Skeletal Muscle Function

After 12 weeks of MCFA intake, mice underwent measurements of submaximal endurance exercise capacity and grip strength [42,43]. Regarding the submaximal endurance exercise capacity testing protocol, seven days before the test, all mice were familiarized with the motorized treadmill (Nazume Co., Ltd., Tokyo, Japan) by running at 15 m/min for 10 min. The test began at 9 m/min for 9 min, then increased to 10 m/min, with an increment of 2.5 m/min every 3 min until reaching 25 m/min. The initial incline was 0°, increasing by 5° every 9 min, with a maximum incline of 15°. Fatigue was the inability to continue running on the treadmill despite repeatedly tapping the mouse’s back to stimulate it. The time of fatigue following running was recorded.

The exercise capacity was expressed in time (min) and work (kg·m). Work performed (kg·m) was calculated as the product of body weight (kg) and vertical distance (meters), where vertical distance = (distance run)(sin θ), where θ is equal to the angle of the treadmill from 0° to 15° [44].

**Figure 12:**
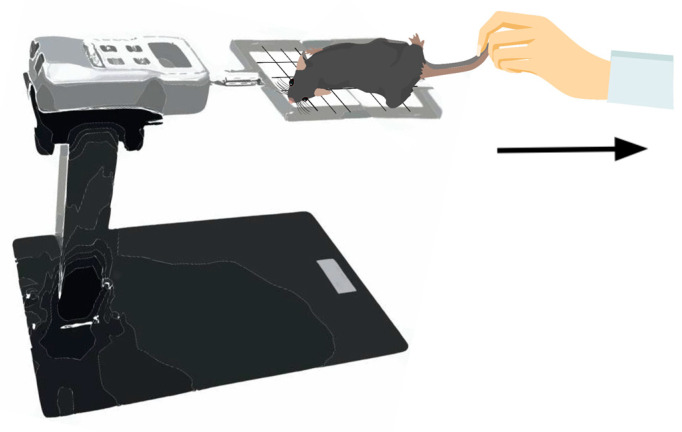
Method of mouse grip strength test. The forelimb grip strength was measured by pulling the mouse horizontally at a constant speed in the direction of the arrow. This figure was drawn using Excalidraw “(https://excalidraw.com/) (accessed on 26 March 2024)”.

Each mouse’s grip strength was measured using a specific grip strength meter (GPM-101BV-C, Melquest, Toyama, Japan). The mouse was placed on a grid connected to the meter. After the animal had grasped the grid, it was pulled horizontally to measure its grip strength. The maximum value from the three measurements was recorded as the maximum grip strength (Figure 12).

**Figure 13:**
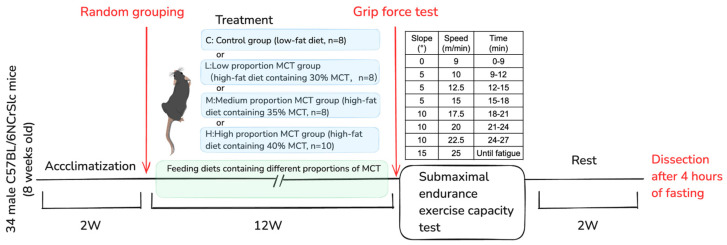
Schematic of the experimental protocol. This figure was drawn using Excalidraw “(https://excalidraw.com/) (accessed on 26 March 2024)”.

Two weeks after the submaximal endurance exercise capacity test and after a 4 h fasting period, mice were lightly anesthetized using inhaled isoflurane (Abbott, Tokyo, Japan) and dissected. Under light anesthesia induced by isoflurane, heparinized blood samples were collected from the abdominal aorta. Various tissues, including the heart, liver, kidney, brown adipose tissue, three white adipose tissues (epididymal, retroperitoneal, and mesenteric), and four skeletal muscles (gastrocnemius, plantaris, soleus, and quadriceps) were excised, weighed, washed with PBS at 4 °C, and frozen in liquid nitrogen. Plasma was obtained from blood samples by centrifugation at 1500× *g* for 10 min at 4 °C. These samples were stored at −80 °C until analyses, as shown in Figure 13.

### 4.4. Real-Time Quantitative Polymerase Chain Reaction (RT-qPCR) for Gene Expression Analysis

Total RNA was extracted from the gastrocnemius and soleus muscles using the TRIzol™ Reagent (Thermo Fisher Scientific Inc., Waltham, MA, USA) according to the manufacturer’s instructions. The concentration and purity of total RNA were assessed using the NanoDrop system (NanoDrop Technologies, Wilmington, DE, USA). Total RNA was reverse-transcribed to cDNA using the High-Capacity cDNA Reverse Transcription Kit (Takara Bio Inc., Shiga Prefecture, Japan) according to the manufacturer’s instructions.

The polymerase chain reaction (PCR) was performed with the Fast 7500 real-time PCR system (Takara Bio Inc., Shiga Prefecture, Japan) using the Fast SYBR ^®^ Green PCR Master Mix (Takara, Kusatsu, Japan). The thermal profiles consisted of 10 min at 95 °C for denaturation followed by 40 cycles of 95 °C for 3 s and annealing at 60 °C for 15 s; 18 s mRNA was used as the housekeeping gene, and the ΔΔCT method was used to quantify target gene expression. All data are represented relative to its expression as fold change based on the values of the L group. PCR primer pairs for each studied gene are shown in Table 4.

### 4.5. Muscle Morphology and Immunohistochemistry

The correct proximal 1/2 of the gastrocnemius muscle tissue was transferred to plastic molds, immersed in optimal cutting temperature compound (Sakura Finetek Japan, Tokyo, Japan), then rapidly frozen by immersion in pre-cooled isopentane and stored at −80 °C. The fixed tissues were cut into 7 μm thick sections using a cryostat. The frozen sections were collected on slides, and the gastrocnemius muscle tissue was stained with hematoxylin and eosin (H&E) to assess muscle cross-sectional area (CSA). ImageJ software (version 1.54p, National Institutes of Health, Bethesda, MD, USA) was used to outline and measure the muscle tissue CSA. The average of 100 randomly selected muscle fiber CSAs for each sample was used for statistical analysis.

### 4.6. Protein Expression Analysis

Total proteins were extracted from the gastrocnemius and soleus muscles using T-PER™ Tissue Protein Extraction Reagent (Thermo Fisher Scientific, Waltham, MA, USA) containing Pierce protease and phosphatase inhibitor mini-tablets, EDTA-free (Thermo Fisher Scientific, Waltham, MA, USA). Protein concentrations were determined using the BCA method, and protein samples were heated at 95 °C for 10 min in a loading buffer containing a 1:9 ratio of 2-mercaptoethanol and 4× Laemmli Sample Buffer (Bio-Rad, Hercules, CA, USA). Equal amounts of protein were loaded onto 10% sodium dodecyl sulfate (SDS)-polyacrylamide gels, separated by electrophoresis, and then transferred to polyvinylidene difluoride (PVDF) membranes (Millipore, Bedford, MA, USA).

After being blocked with a 5% bovine serum albumin (BSA) solution for one hour at room temperature, the membranes were incubated overnight at 4 °C with the specified primary antibodies. Antibodies against total myosin heavy chain (MHC, MF20) were purchased from the Developmental Studies Hybridoma Bank (Iowa City, IA, USA). The above antibodies were used at a 1:2000 dilution. The α-tubulin antibody was purchased from Proteintech (Rosemont, IL, USA) and used at a 1:10,000 dilution.

The membranes were then incubated for 1 h at room temperature with secondary antibodies, either goat anti-rabbit IgG or goat anti-mouse IgG (Cell Signaling Technology, Beverly, MA, USA). The signal was detected using the image reader FujiFilm LAS-300 (FujiFilm, Tokyo, Japan). The grayscale intensity of the protein bands was quantified using ImageJ software (NIH, Bethesda, MD, USA). The phosphorylation levels of mTOR and AMPK were determined by calculating the phosphorylated protein band intensity ratio to the corresponding total protein band intensity. The expression levels of target proteins were normalized to α-tubulin.

### 4.7. Data Analysis and Statistics

All data are shown as mean ± standard deviation (SD). All data were statistically analyzed using GraphPad Prism 10.2 (GraphPad, Ltd., La Jolla, CA, USA). Two-way analysis of variance (ANOVA) and *t*-tests were conducted to determine the main effects of MCFA administration. When this analysis showed significant interactions, simple effects analysis and Tukey’s post hoc tests were performed to assess the significance of means. Additionally, we analyzed the associations between variables using Pearson correlation coefficients. Statistical significance was defined as *p* < 0.05.

## 5. Conclusions

This study shows that a moderate increase in MCFA intake can significantly improve muscle metabolism and function, primarily through upregulating the expression of muscle regulatory factors (MRFs) and myosin heavy chain and inhibiting protein degradation. However, excessive intake may reduce the improvement effects, indicating the importance of moderate consumption. These findings provide new insights into the potential applications of MCFA in improving muscle function and metabolism, emphasizing the necessity of dose control.

## Figures and Tables

**Table 1:** Water and food intake over 12 weeks (g).

		C	L	M	
W1	Water	60.20 ± 5.56	44.18 ± 8.58	41.55 ± 3.78	41.04 ± 4.44
	Feed	49.62 ± 1.16	50.23 ± 0.68	49.81 ± 1.80	51.88 ± 1.49
W2	Water	54.45 ± 3.38	45.98 ± 6.34	40.45 ± 1.89	43.64 ± 2.75
	Feed	48.80 ± 2.71	47.86 ± 2.12	48.46 ± 2.33	51.33 ± 2.24
W3	Water	53.50 ± 1.73	43.35 ± 8.83	41.33 ± 3.02	41.48 ± 11.32
	Feed	49.98 ± 2.33	48.08 ± 1.05	49.00 ± 3.03	48.88 ± 3.34
W4	Water	53.63 ± 4.51	47.18 ± 8.45	46.28 ± 4.36	40.98 ± 5.70
	Feed	47.53 ± 2.32	50.20 ± 1.63	49.45 ± 2.33	47.76 ± 4.38
W5	Water	52.98 ± 6.41	42.6 ± 10.15	41.33 ± 5.87	42.28 ± 7.66
	Feed	47.78 ± 0.85	45.98 ± 0.39	46.78 ± 3.26	46.02 ± 4.85
W6	Water	53.58 ± 3.79	42.35 ± 10.58	42.53 ± 5.36	43.42 ± 7.56
	Feed	47.58 ± 1.64	46.68 ± 1.67	46.20 ± 2.59	46.44 ± 3.90
W7	Water	51.30 ± 4.89	40.73 ± 13.20	36.05 ± 4.25	37.80 ± 6.55
	Feed	46.40 ± 5.12	45.28 ± 6.37	43.23 ± 2.38	45.26 ± 2.78
W8	Water	51.28 ± 4.28	37.65 ± 12.00	35.65 ± 3.84	37.62 ± 5.07
	Feed	47.25 ± 1.86	43.25 ± 1.90	43.35 ± 1.36	43.46 ± 1.72
W9	Water	52.33 ± 1.89	39.80 ± 11.30	36.43 ± 5.51	39.62 ± 3.85
	Feed	48.93 ± 3.29	41.95 ± 3.01	44.10 ± 2.02	44.44 ± 4.90
W10	Water	48.90 ± 4.75	35.53 ± 9.09	35.48 ± 4.08	37.34 ± 9.58
	Feed	47.65 ± 3.90	43.18 ± 4.21	42.25 ± 1.27	44.52 ± 2.94
W11	Water	54.8 ± 3.78	43.23 ± 15.36	38.20 ± 4.28	41.76 ± 6.53
	Feed	47.9 ± 2.91	44.18 ± 3.29	42.85 ± 1.98	44.42 ± 3.80
W12	Water	45.60 ± 4.18	36.10 ± 15.20	31.93 ± 3.01	35.9 ± 4.95
	Feed	46.78 ± 2.77	41.65 ± 3.94	41.90 ± 3.36	41.94 ± 3.44

C group, low-fat diet control group; L group, high-fat diet with low-proportion MCFA (30%, *w*/*w*) treatment group; M group, high-fat diet with medium-proportion MCFA (35%, *w*/*w*) treatment group; and H group, high-fat diet with high-proportion MCFA (40%, *w*/*w*) treatment group. Values are means ± standard deviation (SD).

**Table 2:** Absolute and relative tissue or organ weights.

Name	Unit	C	L	M	H
Heart	mg	136.01 ± 26.34	131.26 ± 17.98	139.63 ± 16.77	143.85 ± 24.72
	%	0.48 ± 0.1	0.41 ± 0.05	0.44 ± 0.08	0.41 ± 0.07
Liver	mg	1159.64 ± 140	1180.29 ± 298.39	1201.26 ± 265.54	1437.27 ± 456.25
	%	4.07 ± 0.48	3.61 ± 0.74	3.72 ± 0.73	3.98 ± 1.04
Kidney	mg	342.51 ± 58.44	341.13 ± 46.96	345.59 ± 48.83	354.51 ± 47.48
	%	1.2 ± 0.2	1.07 ± 0.2	1.08 ± 0.2	1 ± 0.16
Brown fat	mg	111.08 ± 49	192.15 ± 88.08	188.46 ± 60.3	231.31 ± 66.1
	%	0.39 ± 0.17	0.58 ± 0.23	0.57 ± 0.14	0.65 ± 0.17
Epididymal white fat	mg	464.68 ± 150.16	986.6 ± 346.51	919.03 ± 452.01	1217.53 ± 525.89
	%	1.62 ± 0.47	2.98 ± 0.87	2.73 ± 1.04	3.32 ± 1.22
Retroperitoneal fat	mg	133.56 ± 74.17	314.09 ± 178.73	245.04 ± 157.84	355.22 ± 123.55
	%	0.46 ± 0.23	0.94 ± 0.48	0.64 ± 0.44	0.98 ± 0.28
Mesenteric fat	mg	158.27 ± 71.54	317.39 ± 164.01	376.93 ± 207.44	488.72 ± 212.05
	%	0.56 ± 0.25	0.97 ± 0.47	1.12 ± 0.55	1.38 ± 0.63
Gastrocnemius muscle, plantaris muscle, soleus muscle	mg	316.49 ± 20.18	314.46 ± 18.75	322.9 ± 12.42	329.42 ± 16.58
	%	1.11 ± 0.08	0.98 ± 0.09	1.01 ± 0.11	0.93 ± 0.09
Quadriceps muscle	mg	244.68 ± 43.77	215.65 ± 23.94	234.98 ± 20.41	231.58 ± 26.44
	%	0.86 ± 0.15	0.67 ± 0.09	0.74 ± 0.13	0.66 ± 0.09

C group, low-fat diet control group; L group, high-fat diet with low-proportion MCFA (30%, *w*/*w*) treatment group; M group, high-fat diet with medium-proportion MCFA (35%, *w*/*w*) treatment group; H group, high-fat diet with high-proportion MCFA (40%, *w*/*w*) treatment group. Values are means ± standard deviation (SD).

**Table 3:** Composition of experimental diets.

Ingredients (g/100 g)	CON	L, M, and H
Casein	17.2	14.4
L-Cystine	0.26	0.22
Corn starch	30.2	25.2
Maltodextrin	3	2.5
Sucrose	35.8	29.9
Cellulose	4.3	3.6
Test oils	4.3	20
Vitamin mixture	0.86	0.72
Mineral mixture	0.86	0.72
CaHPO_4_	1.1	0.94
CaCO_3_	0.47	0.4
K_3_C_6_H_5_O_7_·H_2_O	1.4	1.2
Choline bitartrate	0.25	0.2

Test oils are cocoa butter. Test oils for L (high-fat diet with low-proportion MCFA (30%, *w*/*w*) treatment group), M (high-fat diet with medium-proportion MCFA (35%, *w*/*w*) treatment group), and H (high-fat diet with high-proportion MCFA (40%, *w*/*w*) treatment group) have different MCFA contents (30, 35, 40%, *w*/*w*).

**Table 4:** Primer sequence for real-time PCR analysis.

Gene	Forward	Reverse
*rps18*	TTCTGGCCAACGGTCTAGACAAC	CCAGTGGTCTTGGTGTGCTGA
*Myod1*	CGCAACGCCATCCGCTACAT	GCATCTGAGTCGCCACTGTAGT
*Myog*	GCAGGCTCAAGAAAGTGAATGA	TAGGCGCTCAATGTACTGGAT
*Myf6*	CCTCAGCCTCCAGCAGTCTTCA	TTACTTCTCCACCACCTCCTCCAC
*Myf5*	CTCAGGAATGCCATCCGCTACA	CCGTCAGAGCAGTTGGAGGT
*Myh4*	GTTCATGCTGACGGATCGTGAGA	AGTCGCCTCCTCCTTCTTCTTGT
*Myh7*	AGATGAATGCCGAGCTCACT	CACATCCAAACCAGCCATCT
*Myh2*	GAGCAAAGATGCAGGGAAAG	TAAGGGTTGACGGTGACACA
*Fbxo32 (ATROGIN1)*	GCTGGATTGGAAGAAGATG	AGAGAATGTGGCAGTGTT
*Trim63 (MuRF1)*	CGACATCTTCCAGGCTGCGAAT	ATCACTTCATGGCGGCACGAG

*rps18*, ribosomal protein S18; *Myod1*, myogenic differentiation 1; *Myog*, myogenin; *Myf6*, myogenic factor 6; *Myf5*, myogenic factor 5; *Myh4*, myosin heavy chain 4; *Myh7*, myosin heavy chain 7; *Myh2*, myosin heavy chain 2; *Fbxo32 (ATROGIN1)*, F-box protein 32; *Trim63 (MuRF1)*, tripartite motif-containing 63.

## Data Availability

Data are contained within this article.

## Supplementary Materials

The following supporting information can be downloaded at https://www.mdpi.com/article/10.3390/ijms26094126/s1.

## Abbreviations

The following abbreviations are used in this manuscript:

MCFAs	medium-chain fatty acids
MCT	medium-chain triglycerides
LCFA	long-chain fatty acids
MRFs	muscle regulatory factors
Myod1	myogenic differentiation 1
Myf6	myogenic factor 6
Myog	myogenin
Myf5	myogenic factor 5
MYH	myosin heavy chain
Myh4	myosin heavy chain 4
Myh7	myosin heavy chain 7
Myh2	myosin heavy chain 2
MHC	myosin heavy chain
Fbxo32	F-box protein 32
Trim63	tripartite motif-containing 63
